# British Hospital and Ambulance Work in France

**Published:** 1916-10-14

**Authors:** 


### BRITISH HOSPITAL AND AMBULANCE WORK IN FRANCE.

### Some Impressions of a Visit.

By A LAY HOSPITAL OFFICER.

(*Continued from p.* 18.)

"Less than two years ago," said a member of the staff of the D.D.M.S. of an important base, "I came here with ten bell-tents, and that was the first hospital accommodation in the neighbourhood." Around where we stood was a group of the familiar neat brown asbestos huts, rows of which were separated by well-kept paths and roads, the whole ground efficiently drained; here and there the grassed corners and borders were relieved by beds of bright flowers. Along a broad valley one saw other groups of huts and many tents, while the adapted hotels and larger buildings of a neighbouring holiday resort are included in the same base, where there are to-day altogether 20,000 beds. And these hospital types, all three sometimes combined in a single unit, are to be found at nearly every base.

The change described is typical of the growth and perfection of the British war hospital system in France. In twenty-four hours the service can efficiently deal with 100,000 casualties, a striking contrast to the distracting days of the autumn of 1914, when patients were arriving in increasing numbers, many with wounds that had not been re-dressed for several days. Then the newer British tourist judged a building fine in so far as it could be converted into a hospital. Any cover, not too unsuitable, was eagerly sought, and stories are told of how in a hotel or casino, turned hospital, the wounded were placed in passages, offices, on the stair-landings, anywhere after the gaming-saloons, concert-halls, and public rooms had been filled to overflowing; and where, in a small operating theatre, ninety-seven operations were carried out within twenty-four hours. To aggravate the situation, through the long day's labour, the insufficient staff was haunted by the fear that work might have to be stopped at any moment and the whole organisation moved fifty miles south. Happily stability of the base was soon assured.

### Faults of Adapted Buildings.

In the absence of better accommodation it is easy to understand how useful these hotels, schools, and other public buildings were in time of stress. To-day, when compared with the hut hospitals, so perfect in their sanitary arrangements, so easily nursed, so saving of labour to stretcher-bearers, so protected against fire risks, it is difficult to follow the arguments for the retention of many of the converted buildings. Much of our hospital accommodation is found in holiday resorts, where buildings have been designed and drainage provided for summer occupancy only. Often the structure is so planned that disaster in the event of the outbreak of fire is more than a possibility; in the absence of a lift, stretcher-work on the staircases must be difficult and hazardous, while a cesspool drainage system, intended perhaps for the use of 150 people for a few months, has to be altered to serve three times as many all the year round. Financial considerations often weigh in questions of adaptation, for when compensation has to be assessed, what is an improvement from the point of view of a hospital will probably be regarded in a different light from that of a hotel. These considerations apart, most of the buildings are very comfortable havens for the patients.

### Huts *versus* Tents.

A hut containing a ward of forty beds and simple offices costs £175, a double tent of the same capacity, £200; the life of the former can hardly yet be estimated, the life of the latter is very brief. Tents, especially in the summer, are comfortable and airy; there is a considerable fire risk, and there are several known cases of fires caused by a carelessly placed cigarette end. The work of the doctor is in some aspects not easy in a tent. "When there is a wind one cannot listen to a chest for the noise of the flapping canvas and the humming amongst the ropes." But the possibilities of tents for hospital purposes are only grasped when one sees five marquees, end on end, containing seventy beds—a great ward under whatever roof. One hospital is the possessor of a number of sumptuous tents, originally used in the Durbar, and presented for war purposes by a Raja. These are used for officers, and though containing few beds are quiet and extremely comfortable.

One is struck by the extreme simplicity and economy of the equipment of all the Government hospitals and most of the other institutions. The *x*-ray outfit is generally the only feature which has cost an important sum. The R.A.M.C. beds are of trestle type, elsewhere the more labour-saving caster bed is in use. The easily packed and transported trestle bed is doubtless part of the theoretic equipment of a hospital moving its position from time to time.

### Base Special Hospitals.

In several instances a hospital has been recognised as the centre to which certain cases are to be sent. To one will come most of the ghastly face wounds for skilful special treatment, which in a wonderfully short time will effect such surprising changes in the appearance of the patients; to another are taken cases of blindness and severe head wounds which often cause blindness, elsewhere will be found mental cases and those of shell shock. One unit with 1,000 beds in huts, tents, and bathing sheds receives enteric and other infectious cases. Happily, the full accommodation of this hospital is not always in use, for infectious disease in this war has been greatly limited by the burning of all fæcal matter. In this enteric hospital there is a splendid pathological department and an excellent system of records, each case being described with exhaustive particulars of the results of tests and other observations.

